# Flame Retardants in Placenta and Breast Milk and Cryptorchidism in Newborn Boys

**DOI:** 10.1289/ehp.9924

**Published:** 2007-05-31

**Authors:** Katharina Maria Main, Hannu Kiviranta, Helena Eeva Virtanen, Erno Sundqvist, Jouni Tapio Tuomisto, Jouko Tuomisto, Terttu Vartiainen, Niels Erik Skakkebæk, Jorma Toppari

**Affiliations:** 1 University Department of Growth and Reproduction, Rigshospitalet, Copenhagen, Denmark; 2 National Public Health Institute, Department of Environmental Health, Kuopio, Finland; 3 Departments of Physiology and Paediatrics, University of Turku, Turku, Finland; 4 University of Kuopio, Department of Environmental Sciences, Kuopio, Finland

**Keywords:** breast milk, cryptorchidism, exposure, human, infant, polybrominated diphenyl ethers

## Abstract

**Background:**

Polybrominated diphenyl ethers (PBDEs) are widely used in Western countries.

**Objectives:**

Because the prevalence of cryptorchidism appears to be increasing, we investigated whether exposure to PBDEs was associated with testicular maldescent.

**Methods:**

In a prospective Danish–Finnish study, 1997–2001, all boys were examined for cryptorchidism. We analyzed whole placentas (for 95 cryptorchid/185 healthy boys) and individual breast milk samples (62/68) for 14 PBDEs and infant serum samples for gonadotropins, sex-hormone binding globulin, testosterone, and inhibin B.

**Results:**

In 86 placenta–milk pairs, placenta PBDE concentrations in fat were lower than in breast milk, and a larger number of congeners were nondetectable. There was no significant difference between boys with and without cryptorchidism for individual congeners, the sum of 5 most prevalent, or all 14 congeners. The concentration of PBDEs in breast milk was significantly higher in boys with cryptorchidism than in controls (sum of BDEs 47, 153, 99, 100, 28, 66, and 154: median, 4.16 vs. 3.16 ng/g fat; *p* < 0.007). There was a positive correlation between the sum of PBDEs and serum luteinizing hormone (*p* < 0.033). The sum of PBDEs in breast milk did not differ between Denmark and Finland (median, 3.52 vs. 3.44 ng/g fat), but significant differences in some individual congeners were found.

**Conclusions:**

Two different proxies were used for prenatal PBDE exposure, and levels in breast milk, but not in placenta, showed an association with congenital cryptorchidism. Other environmental factors may contribute to cryptorchidism. Our observations are of concern because human exposure to PBDEs is high in some geographic areas.

Polybrominated diphenyl ethers (PBDEs) are widely used as flame retardants, and the general population is exposed through products such as upholstery, building materials, insulation, electronic equipment, and contaminated food. PBDEs are added to polymers without being chemically bound and can leach into the environment, where they settle with air particles and sludge. They are persistent, and some—BDE-47, BDE-99, and BDE-153—can accumulate in lipid-rich tissues (Agency for Toxic Substances and Disease Registry 2004; Sjödin et al. 2003).

Concentrations of PBDE in human European breast milk samples are generally low compared with those in the United States, and considered to be well below the estimated lowest observed adverse effect level (LOAEL) of 1 mg/kg/day (Darnerud et al. 2001). Two technical mixtures, penta- and octa-mixtures of PBDEs, have been banned from use in Europe since 2003 (Darnerud et al. 2001), and Swedish studies indicated a decrease in breast milk levels since the middle of the 1990s (Meironyte et al.1999; Sjödin et al. 2003). However, annual production rates of some PBDEs are still considerable in some areas (Alaee et al. 2006; Betts 2002; Law et al. 2006). Animal studies show that some PBDEs exhibit endocrine-disrupting activity, which has been studied predominantly for thyroid hormone transport and metabolism (Legler and Brouwer 2003), but data on adverse effects on reproductive outcome after gestational exposure are also emerging (Lilienthal et al. 2006).

The prevalence of cryptorchidism in newborn boys appears to have increased in some areas, such as Great Britain and Denmark, over the past decades, and its current prevalence is considerably higher in Denmark than in Finland (Anonymous 1986; Boisen et al. 2004). Although the reason for this is as yet unknown, the rapid increase in prevalence suggests that environmental factors are involved (Sharpe 2006; Skakkebæk et al. 2001). Adverse effects of fetal exposure to environmental chemicals on testicular descent and hormonal function may be detectable during the short physiologic activation of the pituitary–gonadal axis at approximately 3 months of age (Andersson et al. 1998; Main et al. 2000, 2006b; Suomi et al. 2006).

In this study we aimed to evaluate the association between exposure to 14 PBDEs (BDEs 28, 47, 66, 71, 75, 77, 85, 99, 100,119, 138, 153, 154, 183) in newborn boys and the position and function of the testes.

## Materials and Methods

The study was conducted according to the Helsinki II Declaration (World Medical Association 2000) after informed oral and written consent of the parents. The ethical committees (Finland: 7/1996; Denmark: KF01-030/97) and the Danish Data Protection Agency (1997-1200-074) approved the study.

### Study population

We obtained breast milk samples and placentas from a joint prospective, longitudinal cohort study performed 1997–2001 at Turku University Hospital, Turku, Finland, and the National University Hospital (Rigshospitalet, Hvidovre Hospital), Copenhagen, Denmark. This binational study aimed at establishing contemporary prevalence rates for cryptorchidism and hypospadias and evaluating risk factors by means of questionnaires and biological samples (blood, placentas, breast milk). Exposure measurements were prospectively planned to include persistent and nonpersistent chemicals, some of which have been previously reported (Damgaard et al. 2006; Main et al. 2006a; Shen et al. 2005, 2006, 2007). Recruitment strategy, inclusion criteria, and clinical examination of the children (i.e., the identification of cryptorchidism) have been previously described (Boisen et al. 2004; Main et al. 2006b; Suomi et al. 2006) and were strictly standardized. Boys with normally descended testes, including retractile testes, were used as controls in this study under the terms “controls” or “healthy boys.” Boys with undescended testes (nonpalpable, inguinal, suprascrotal, high scrotal), either uni- or bilaterally at birth, were included in the group of cryptorchid boys.

All boys were examined at birth and at 3 months of age before the results of chemical analyses were known. Birth weight and length were obtained from hospital records. The supine length of the children was measured with infantometers (Denmark: Kidimeter, Raven Equipment Ltd., Essex, United Kingdom; Finland: Pedihealth Ky, Oulu, Finland). Weight was measured on a digital scale (Baby scale model; Solotop Oy, Helsinki, Finland). Weight for gestational age was calculated using national standards as percent deviation from the expected mean (Marsál et al. 1996; Pihkala et al. 1989), −22% being equivalent to −2 SDs.

### Biological samples

In Denmark biological samples were collected from all participants (case–cohort design). In Finland, due to lack of storage space, biological samples were collected from boys with cryptorchidism at birth and matched controls [matching criteria: parity, maternal smoking (yes/no), diabetes (yes/no), gestational age (± 7 days), and date of birth (± 14 days)] as a nested case–control design.

From this bio-bank, we selected 280 placentas (168 Danish/112 Finnish) and 130 breast milk samples (65 Danish/65 Finnish) for PBDE measurements; this number was determined by funding. Birth examination data were used for classification of cryptorchid and healthy boys.

In Finland, placentas were selected from 56 case–control pairs, in which both placentas were available. In Denmark, all available placentas from cryptorchid boys were chosen (*n* = 39). Control placentas were selected randomly from the total Danish cohort (*n* = 129).

Hereafter, 65 breast milk samples were selected for each country, with the aims, if possible, of having equal numbers of samples from boys with and without cryptorchidism and of including the same mothers as for placenta analyses. Only samples with a volume > 125 mL were included to ensure that all chemicals could be analysed. Milk from mothers of 29 cryptorchid boys and 36 randomly chosen control boys was included in Denmark. In Finland, milk samples were chosen from mothers of cryptorchid boys (*n* = 33), matched controls (*n* = 18), or random controls (*n* = 14).

For 86 boys, milk and placentas could be selected from the same mother–child pair (10 Danish boys with cryptorchidism, 33 Danish controls, 20 Finnish boys with cryptorchidism, 23 Finnish controls).

### Collection of samples

Whole placentas were collected by the midwives and frozen immediately in two layers of polyethylene bags (−20°C). Placentas were not bled before storage.

Each mother collected one breast milk sample. We wished to assess the average concentration of PBDEs during the period preceding the endogenous hormone surge in infants. Thus, each sample consisted of several small aliquots collected over successive feedings over several weeks and frozen in household freezers in 250-mL Pyrex glass bottles (1515/06D; Bibby Sterilin, Staffordshire, UK) with Teflon coated caps. The mothers were instructed orally and in writing to feed the baby, and then to sample aliquots (hind milk), beginning 1 month after birth. We chose this start point after discussion with the ethics committee for human subject studies to ensure that breast-feeding had been well established. Mothers were instructed to collect samples into a clean household glass or porcelain container, avoiding, if possible, the use of breast pumps, and to freeze every portion immediately. Breast milk was delivered frozen to the hospital at the 3 months’ examination and stored at −20°C. In 57 of the 65 Danish mothers, but no Finnish mothers, information on breast pump use was obtained at sample delivery; 26 (46%) had used a pump on one or more occasions. No information was obtained on the type of breast pump (glass or plastic).

### Blood samples

Venous nonfasting blood samples (4 mL) were drawn from the infants at the 3 months’ examination (median age, 3.0 months; range, 2.4–4.1); the overall success rate of obtaining a sample in the study was 74%. After clotting, the blood samples were centrifuged and the sera were separated and stored at −20°C. All samples were analysed as duplicates and blinded for the technician at one laboratory (Rigshospitalet, Denmark) for reproductive hormones. Each run contained samples of both cryptorchid and healthy boys from both countries (up to 160 samples per analysis) to minimize any effect of interassay variation.

### Hormone analyses

We analyzed serum follicle-stimulating hormone (FSH), luteinizing hormone (LH), and sex hormone–binding globulin (SHBG) using time-resolved immunofluorometric assays (Delfia, Wallac Inc., Turku, Finland). Detection limits were 0.06 and 0.05 IU/L for FSH and LH, respectively, and 0.23 nmol/L for SHBG. The intra- and interassay coefficients of variation (CV) were < 5% in both gonadotropin assays and < 6% for SHBG. We measured serum testosterone by radioimmunoassay (Coat-a-Count; Diagnostic Products Corp., Los Angeles, CA, USA), with a detection limit of 0.23 nmol/L and intra- and interassay CVs < 10%. Free testosterone index was calculated: (testosterone × 100)/SHBG. We analyzed serum inhibin B by a double antibody enzyme-immunometric assay (Main et al. 2006b). The detection limit was 20 pg/mL, and intra- and interassay CVs were < 15 % and < 18 %, respectively. Ratios between hormones were calculated: LH/testosterone, LH/free testosterone, FSH/inhibin B.

### Analysis of PBDE

All PBDE analyses for both milk and placenta were performed at the laboratory at the Department of Environmental Health in Kuopio, Finland. Placentas were defrosted, and the umbilical cord and all readily removable membranes were discarded. Whole placentas were homogenized in a mixer (Büchi Mixer B-400; Büchi Laboratories AG, Flawil, Switzerland), and 75 g of the homogenate was lyophilized. Dried homogenate was pulverized in a mortar and slurry was made by adding dichloromethane and cyclohexane (1:1 vol/vol) and concentrated sulphuric acid. This slurry was spiked with six ^13^C-labeled PBDE internal standards (BDEs 28, 47, 77, 99, 153, and 183) (Wellington Laboratories Inc., Guelph, Canada). Fat was not determined, and fat-based results were relying on the gravimetrically measured fat contents obtained from a German partner in this European Commission project (Shen et al. 2005, 2006, 2007).

Breast milk samples (average volume, 70 mL) were thawed in sample bottles in a water bath (40°C) for 1 hr and homogenized. Fat was extracted with a mixture of diethyl ether and hexane (1:1.4 vol/vol) after addition of sodium oxalate solution and ethanol (1:5 vol/vol). Fat content was determined gravimetrically after exchange of the solvent to hexane. An average 1.5 g of fat was spiked with the same set of internal standards used with placenta.

The procedure for decomposition of fat and sample cleanup has been described previously (Kiviranta et al. 2004). We quantified 14 PBDE analytes (BDEs 28, 75, 71, 47, 66, 77, 100, 119, 99, 85, 154, 153, 138, and 183) by selective-ion recording using a high resolution mass spectrometer (Autospec Ultima; Micromass Inc., Manchester, UK) at resolution 10,000. Gas chromatographic separation of the PBDEs was performed with a Hewlett Packard 6890 gas chromatograph with fused silica capillary column (DB5-MS, 60 m, 0.25 mm, 0.25 μm; J&W Scientific, Folsom, CA, USA). As a recovery standard for internal PBDE standards polychlorinated biphenyl congener 159 was used.

In the analysis of PBDEs at the Department of Environmental Health (National Public Health Institute, Kuopio, Finland), the technicians and chemists were blinded. Laboratory and cross-sample contamination was monitored by analyzing procedural blank samples. The concentrations of these blank samples were much lower than the concentrations in placenta and breast milk—on average, 3.6 and 2.6% of the average sum of PBDEs in placenta and milk, respectively.

Recoveries of individual internal PBDE standards were > 60%. Median limit of quantification (LOQ) for placentas corresponding to a signal to noise ratio of 3:1, was 0.006 ng/g fat (range, 0.004–0.14 ng/g fat). Corresponding LOQs for breast milk were 0.004 ng/g fat (range, 0.0003–0.12 ng/g fat). In placenta, CVs for individual congeners were 10–20%, and > 20% at concentration levels 0.1–1 ng/g fat and < 0.1 ng/g fat, respectively. In breast milk samples corresponding values were < 10%, 10–20%, and > 20% at concentrations levels > 1 ng/g fat, 0.1–1 ng/g fat, and < 0.1 ng/g fat, respectively. Concentrations below the LOQ were considered to be equal to nil (lower bound results). The laboratory has successfully participated in interlaboratory comparison studies of PBDEs in different biological matrices including breast milk (Becher et al. 2001; Småstuen and Becher 2004; Småstuen and Becher 2005). The Finnish Accreditation Service (FINAS; Espoo, Finland) has verified the competence of the laboratory (testing laboratory T077) in performing PBDE analyses in biological samples according to the European Standard (EN ISO/IEC 17025).

## Statistics

Population characteristics are given as medians and percentiles (2.5th, 97.5th). Differences between boys with and without cryptorchidism and between Danish and Finnish populations were analysed by Mann-Whitney *U*-test. Eighty-six boys participated with both breast milk and placenta samples.

We tested country differences for (log-transformed) PBDE concentrations in breast milk and placenta by multiple linear regression including maternal age, parity (1, 2 and ≥ 3) and prepregnancy body mass index (BMI; kilograms per square meter) in the model.

Correlations between individual PBDE congener concentrations, and between PBDE concentrations and date of childbirth within the Danish and Finnish cohort, respectively, were tested by Spearman correlation on non-transformed data. For each country the date of birth for the first child was set at zero; the date of birth for all consecutive children was then calculated as number of days elapsed since the first child of the same country-specific cohort. This variable was applied to control for any time trends in flame-retardant concentration in the study period 1997–2001.

We tested differences in PBDE concentrations between boys with and without cryptorchidism in a multiple regression model, including as covariates maternal age, parity, maternal prepregnancy BMI, and date of childbirth within the cohort (days) to control for factors that could affect PBDE concentrations in the sample. Prematurity and small size for gestational age (SGA) are well-known risk factors for cryptorchidism. Because the number of premature (5 Danish, 3 Finnish) and SGA children (3 Danish, 1 Finnish) was small in this study, analyses were carried out both with and without inclusion of these parameters. Analyses were carried out only for the most abundant seven congeners in breast milk (and their sum) and for the most abundant five congeners in placenta.

We used multiple linear regression analysis to assess the correlation between serum levels of reproductive hormones (log-transformed LH, FSH, SHBG, inhibin B), square root–transformed serum testosterone or free testosterone index, and log-transformed PBDE concentrations in breast milk. Covariates included in these analyses were country of origin, testicular position (cryptorchidism/control), and age at blood sampling (months).

## Results

There were no significant differences in maternal age, reported smoking, and parity between cryptorchid and control boys (Tables 1 and 2). Gestational age and birth weight were slightly lower in cryptorchid Danish (but not Finnish) boys than in controls. Diabetes was more prevalent in Finnish (but not Danish) mothers of cryptorchid boys than in their controls, because we could not always find controls matched by this criterion. The date of childbirth within the study period did not differ significantly between cryptorchid boys and controls (Denmark, *p* = 0.327; Finland, *p* = 0.949).

Danish mothers were slightly older than Finnish [30.6 years; 95% confidence interval (CI), 23.6–38.8 vs. 28.7 years; 95% CI, 21.4–39.7; *p* < 0.011], had a lower parity (*p* < 0.033) and smoked more frequently (*p* < 0.04). The prevalence of diabetes mellitus was higher among Finnish women (*p* < 0.004), but BMI before pregnancy (*p* = 0.678) did not differ significantly between the countries (*p* = 0.225).

### Breast milk

**Figure 1:**
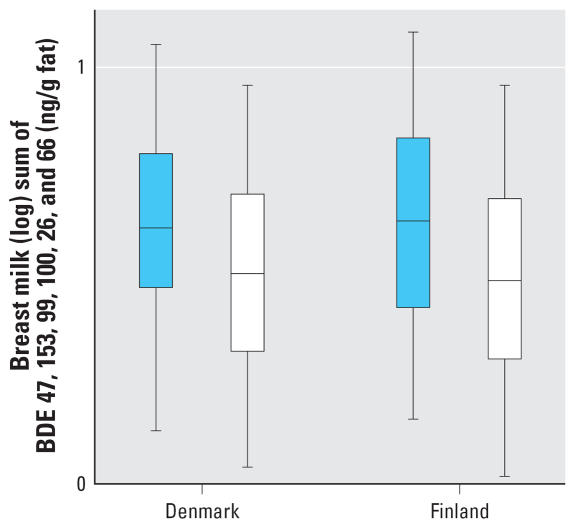
Sum of the 7 most prevalent PBDEs in breast milk samples (BDEs 47, 153, 99, 100, 28, 66, 154, log-transformed values) from Denmark and Finland in boys with cryptorchidism (*n* = 62, blue) and healthy boys (*n* = 68, white). The box plot shows medians and interquartile ranges.

Seven PBDEs were measurable in all breast milk samples (Table 3). Median concentrations were higher in Danish but not Finnish cryptorchid boys than in controls, reaching statistical significance for BDEs 47, 100, 28, 66, and 154. The sum of all seven congeners was significantly higher in cryptorchid boys than in controls if both countries were analyzed together (*p* < 0.007) (Figure 1), also if prematurity and SGA were included in the model (*p* < 0.035). Similar results were obtained for PBDE expressed as nanograms per liter (data shown only for the sum of all 14 congeners; Table 3). Table 4 shows the remaining seven congeners, which were below the detection limit in a substantial number of samples, and the sum of all 14 congeners.

**Figure 2:**
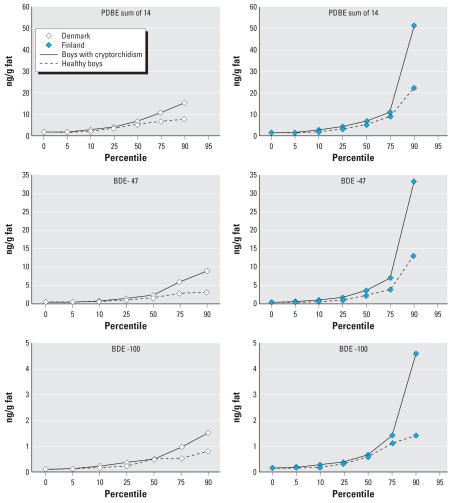
Concentration (percentiles) of the sum of all PBDEs, BDE-47, and BDE-100 (ng/g fat) in human breast milk samples from Denmark (*n* = 65) and Finland (*n* = 65), 1997–2001. Note the differences in absolute values in the *y*-axis.

The concentrations of PBDE congeners in milk samples showed large variations between congeners and individuals (Figure 2). Individual congener concentrations were positively correlated with one another (*r* = 0.178–0.955, *p* < 0.0001).

There were no significant country differences in the sum of all congeners [Denmark, 3.52 ng/g (95% CI, 1.26–14.2) vs. Finland, 3.44 ng/g (95% CI, 1.25–13.94), *p* = 0.754] or the sum of the most prevalent seven congeners, BDEs 47, 153, 99, 100, 28, 66, and 154 [Denmark, 3.24 ng/g (95% CI, 1.26–51.1) vs. Finland, 3.23 ng/g (95% CI, 1.26–51.0), *p* = 0.629]. Similar results were obtained if we analyzed only milk samples from mothers of healthy boys (data not shown). The estimated daily intake of PBDEs (sum of all) for an infant at 3 months of age (median infant body weight 6.58 kg, consuming 120 mL breast milk/kg) was a median of 16 ng/kg/day (range, 6–121 ng/kg/day). Breast milk lipid (percent wet weight) differed significantly between the countries (mean ± SD = 2.99 ± 1.38 in Denmark, 4.52 ± 1.56 in Finland; *p* < 0.0001). The lipid content was not significantly correlated with the sum of PBDE congeners (data not shown).

The pattern of congener distribution differed between the countries. BDE-28 was significantly higher in Finland than in Denmark (*p* < 0.021), with a similar tendency for BDE-47 (*p* < 0.077), which was the most prevalent congener. BDE-153 (*p* < 0.0001), BDE-66 (*p* < 0.026), and BDE-183 (*p* < 0.022) were significantly higher in Denmark than in Finland, with a similar tendency for BDE-99 (*p* < 0.073). For congeners at very low concentrations, the percentage of measurable samples was higher in Denmark than in Finland (Table 4). There was no significant effect of breast pump use during collection of samples on the concentration of any BDE congener in the Danish breast milk samples.

In the Danish but not the Finnish samples, the date of childbirth within the cohort was significantly correlated with the concentration of BDE-154 (*r* = −0.346, *p* < 0.005), BDE-85 (*r* = −0.434, *p* < 0.0001) and BDE-75 (*r* = 0.376, *p* < 0.002) with similar tendencies for BDE-66 (*r* = −0.211, *p* = 0.092) and BDE-77 (*r* = −0.214, *p* = 0.087).

Serum LH levels correlated positively with the sum of seven PBDEs in breast milk (*r* = 0.218, *p* < 0.033) as well as with individual congeners BDE-47 (*r* = 0.227, *p* < 0.027), BDE-100 (*r* = 0.293, *p* < 0.004), and BDE-154 (*r* = 0.203, *p* < 0.048). Country-specific analyses showed significant associations between serum LH and the above listed PBDEs and their sum for Finnish milk samples, but not Danish (data not shown). No other reproductive hormones or their ratios were significantly correlated with the concentration of PBDEs.

### Placentas

The average levels of 14 PBDE congeners per gram fat in placentas were lower than in breast milk (Table 5), and more samples were nondetectable. Therefore, the sum of five congeners was used instead of the seven congeners used for milk. This had a minor influence on the total, because the sums of the five and the 14 congeners were very close.

The distribution of congeners resembled the distribution in breast milk, with BDE-47 and BDE-153 constituting the main fraction of PBDEs. There was no significant country difference for the sum of all 14 congeners (*p* = 0.198) or the sum of five (BDE-47, BDE-153, BDE-99, BDE-100, and BDE-28; *p* = 0.192). This was also true when only placentas from healthy boys were analyzed (data not shown). The concentrations of the five most prevalent congeners were positively correlated with each other (*r* = 0.171–0.827, *p* < 0.0001). Placenta lipid content (percent wet weight) differed significantly between the countries (mean ± SD = 1.09 ± 0.17 in Denmark, 1.21 ± 0.13 in Finland; *p* < 0.0001). Placenta lipid content was not significantly correlated with the sum of PBDE congeners (data not shown).

The date of childbirth within the cohort was significantly correlated in the Danish placentas with the concentration of BDE-66 (*r* = −0.246, *p* < 0.001), and in the Finnish placentas with BDE-85 (*r* = 0.239, *p* < 0.01) and BDE-153 (*r* = 0.275, *p* < 0.003).

There was no significant difference in the placenta concentration of the five most prevalent PBDEs between cryptorchid boys and healthy boys in Denmark (*p* = 0.10–0.976) or Finland (*p* = 0.09–0.835) or for their sum (*p* = 0.312 and *p* = 0.128, respectively). The results remained nonsignificant if prematurity and SGA were included in the model. There were no correlations between placental PBDEs and serum reproductive hormones in 3-month-old infants.

### Paired samples

The median concentrations (nanograms per gram fat) of the 14 congeners in placenta were lower than the concentration found in the paired breast milk samples (*n* = 86) (Table 6), but there were significant correlations between the measurements, except for BDE-85 and BDE-138.

The sum of all congeners in milk was 3.39 (95% CI, 1.43–48.2) for boys with cryptorchidism and 3.15 (95% CI, 1.07–24.9) for controls (*p* = 0.228), and in placenta 1.22 (95% CI, 0.64–9.32) for boys with cryptorchidism and 1.17 (95% CI, 0.49–5.46) for controls (*p* = 0.871). Infant reproductive hormones at 3 months of age (27 Danish, 35 Finnish boys) were not correlated to PBDE concentrations in placenta or milk in this data subset.

## Discussion

To our knowledge, this is the first study describing an association between congenital cryptorchidism in humans and exposure to PBDEs. An association was found for the sum of seven PBDEs (BDEs 47, 153, 99, 100, 28, 66, 154) in breast milk as well as for the individual congeners BDEs 47, 100, 28, 66, and 154. Concentrations of BDEs 47, 100, and 154 were also positively correlated with increasing serum LH values. This suggested that a higher gonadotropin drive was necessary to ensure normal testosterone production by the Leydig cells (Suomi et al. 2006), and thus a subtle primary testicular disfunction.

In this study we assessed infant exposure by measuring the concentration of PBDEs in breast milk, which reflects the accumulated body burden of the mother (Inoue et al. 2006; Jensen and Slorach 1991; Waliszewski et al. 2001). It also is a proxy for prenatal fetal exposure because PBDEs, especially the lower brominated compounds, can cross the placenta (Bi et al. 2006; Mazdai et al. 2003) and are transferred to breast milk during lactation (Darnerud and Risberg 2006). However, when we assesssed exposure by measuring PBDE in placenta, the results did not support the above findings, despite the fact that 86 mother–child pairs were represented with both milk and placenta samples. At present, it is not clear why this is the case.

The concentration of all PBDE congeners per gram fat in placenta was considerably lower than in milk, and more congeners were nondetectable. These differences did not have any remarkable influence on analytical errors, because the amount of fat used for analysis was similar for placentas and milk samples. In theory, placenta should be a better proxy for fetal exposure than breast milk, because it represents the direct route of chemical transfer during pregnancy. The number of placentas analyzed in this study was larger than the number of milk samples, and should thus better represent the pool of cases and controls. We could not establish any obvious selection bias of milk donors. On the other hand, due to higher concentrations, milk analyses were somewhat more reliable toward the lower end of concentration. This should not be important, because the main group differences were seen at the higher end of PBDE concentrations. It is currently unknown whether PBDEs accumulate in placenta, but our paired samples indicated that this may not be the case. We found positive correlations between measurements in placenta and breast milk, but three to four times lower absolute concentrations in placenta. Placenta concentrations may resemble measurements in single blood samples, and thus reflect the situation at delivery but not the long-term exposure.

There is some controversy as to whether the placental transport of PBDEs may differ between lower and higher brominated PBDEs. An American study reported a strong correlation of lipid-adjusted BDE concentrations in maternal serum at term and cord blood (Mazdai et al. 2003), whereas studies from countries with a generally lower exposure level found weaker correlations and higher values in maternal than in fetal samples (Bi et al. 2006). Segregation from serum into breast milk samples did not always follow a 1:1 pattern. The congener distribution appeared to be similar for lower brominated BDEs (Bi et al. 2006; Mazdai et al. 2003), whereas higher brominated BDEs such as BDE-209 were 10 times higher in serum than in breast milk (Inoue et al. 2006). Thus, the relative distribution of congeners between placenta and breast milk may also depend on the fat composition of these two matrices causing different solubility. We found significantly higher fat concentrations in the Finnish than in the Danish samples, which may reflect dietary habits in the two countries, because both long-term and short-term diet as well as the nutritional status may influence content and composition of breast milk lipids (Ortiz-Olaya et al.1996).

Current knowledge about human reproductive health consequences after exposure to PBDEs is very limited (Agency for Toxic Substances and Disease Registry 2004). A study from Taiwan showed an association between the sum of 12 PBDE congeners in breast milk, 10 of which were the same as in our study, and lower birth weight and length (Chao et al. 2007). Recently, a Swedish case–control study found higher values of PBDEs (sum of BDEs 47, 153, and 99) in blood samples from mothers of young men with testicular cancer than in age-matched controls (Hardell et al. 2006). However, maternal PBDE exposure was assessed 15–25 years after the critical time period—that is, at the time of the cancer diagnosis—which considerably weakens the possibility to establish a causal link between exposure and outcome. Also in this study (Hardell et al. 2006), exposures to other persistent chemicals occurred simultaneously, and it cannot be determined how these substances interact in their effect on reproductive development. Testicular cancer is the most severe clinical symptom of the testicular dysgenesis syndrome (TDS), which also encompasses impaired semen quality, congenital cryptorchidism, and hypospadias (Skakkebæk et al. 2001). TDS may be caused by genetic, hormonal, or environmental factors (Sharpe 2006; Skakkebæk et al. 2001). Prenatal exposure to PBDEs may have an adverse effect on testicular growth and differentiation *in utero*.

In animal studies, penta-brominated PBDEs showed antiandrogenic activity (Stoker et al. 2005). A peripubertal single-dose exposure to a commercial mixture of penta-BDEs delayed the onset of puberty in male and female rats (Stoker et al. 2004) and suppressed the growth of the seminal vesicles and ventral prostate. In adult rats, a single-dose exposure significantly increased LH concentration in serum (Stoker et al. 2005). Our observation of an association between PBDE levels in breast milk and serum LH in infants at 3 months of age is in line with these animal data. Gestational exposure of rats to BDE-99 caused a shortening of the anogenital distance in male and female rats, reduction in primordial and secondary ovarian follicles, a lower sperm count, and lower estradiol and testosterone levels in adulthood (Kuriyama et al. 2005; Lilienthal et al. 2006). *In vitro* assays showed a competitive androgen receptor binding for BDEs 47, 99, and 100. BDEs 47, 71, and 100 inhibited dihydrotestosterone-induced transcriptional activity (Stoker et al. 2005). Because testicular descent is highly androgen-dependent (Toppari 2003), the adverse effect of PBDE on testicular descent could be caused by their antiandrogenic properties.

In addition, BDEs 47, 100, 75, and 51, particularly their hydroxylated metabolites, are weakly estrogenic, and BDEs 153, 166, and 190 are antiestrogenic *in vitro* (Legler and Brouwer 2003; Meerts et al. 2001). Exposure of female rats to BDE-99 led to the formation of abundant vesicles and vacuolization of the ovary (Talsness et al. 2005), which showed signs of compromised steroidogenesis. Prenatal female exposure to BDE-99 in rats affected estrogen target genes in the uterus (Ceccatelli et al. 2006). Thus, the delicate balance between androgens and estrogens in the fetus may become altered by PBDE exposure. In mice, the metabolism of BDE-47 was highly dependent on the developmental stage of the animal, being slowest in pups (Staskal et al. 2006). Whether this also plays a role for human fetal development is currently unknown. Most exposure doses used in animal studies are several orders of magnitude higher than the levels of PBDEs found in breast milk in our study. However, there is emerging evidence that also low-dose exposure to, for example, BDE-99 close to levels found in human adipose tissue may have an adverse effect on the reproductive health of the offspring (Anonymous 2005; Kuriyama et al. 2005).

The distribution pattern of BDE congeners in breast milk corresponded to the distribution of BDE congeners in commercially available mixtures of PBDEs (Alaee et al. 2006; Darnerud et al. 2001; Law et al. 2006; Lilienthal et al. 2006). The absolute concentrations found in our study are within the same order of magnitude as reported from other Nordic and European countries (Jaraczewska et al. 2006; Kalantzi et al. 2004; Lind et al. 2003; Strandman et al. 2000) as well as China (Bi et al. 2006) and Japan (Eslami et al. 2006; Inoue et al. 2006). There are, however, significant geographic differences between and within these countries, which point toward differences in general contamination levels. In our study, the total amount of PBDEs did not differ between Finnish and Danish milk or placenta samples, but the pattern of congener distribution varied, which indicated different sources and timing of exposure. PBDE levels reported from American studies of breast milk appear to be considerably higher (Betts 2002). In contrast, the previous exponential increase in penta-PBDEs in Swedish breast milk samples since the 1970s has reversed since the late 1990s (Law et al. 2006; Meironyte et al. 1999; Sjödin et al. 2003), when penta-BDEs were gradually phased out. The collection of breast milk samples in our study covered a 5-year period, and we found a negative correlation between the level of PBDEs in breast milk and the infant date of birth. This is in line with the expected decline in the use of penta-PBDEs in the two regions. The high variability in PBDE concentrations among individual mothers has been described also for other human matrices (McDonald 2002), and may reflect variability in both exposure and metabolism. Deca-BDE can be converted through sunlight exposure into lower brominated BDEs, which are more readily absorbed from the intestine and bioaccumulate due to their longer half-life than deca-BDE (Watanabe and Tatsukawa 1987). However, it is as yet unknown how much this process contributes to human environmental exposure to lower brominated PBDEs.

Exposure to PBDEs cannot explain the observed geographic difference in the prevalence of cryptorchidism between Denmark and Finland. Breast milk contains significant levels of other persistent and nonpersistent chemical compounds (Damgaard et al. 2006; Main et al. 2006a), which can affect perinatal testicular development. Mothers with high levels of PBDE exposure also may be exposed to high levels of other persistent chemicals. Thus, the combined exposure to multiple environmental factors may cause the association between congenital cryptorchidism and PBDE concentration in breast milk (Koppe et al. 2006). In addition, other lifestyle factors and genetic susceptibility may play a role (Damgaard et al. 2007; Sharpe 2006; Virtanen et al. 2006). In our total study population, the geographic difference in the prevalence of cryptorchidism was observed mainly for mild forms of undescended testes, which had a high degree of spontaneous postnatal descent (Boisen et al. 2004). This pattern was also seen in this subpopulation, in which PBDEs was analyzed. However, also mild and transient forms of cryptorchidism are associated with a subtle impairment of primary testicular function (Suomi et al. 2006).

In conclusion, we used two different biological matrices in this study to assess infant perinatal exposure. Breast milk, but not placenta, showed an association with congenital cryptorchidism. There are valid arguments for either matrix, and risk assessment will require more scrutiny. The association between PBDE contamination levels in breast milk and congenital cryptorchidism is still of concern because exposure to PBDEs is considerable in some areas.

## Figures and Tables

**Table 1:** Population characteristics [median (2.5th–97.5th percentiles)] for breast milk samples.

	Denmark			Finland		
Characteristic	Control (*n* = 36)	Cryptorchid (*n* = 29)	*p*-Value*	Control (*n* = 32)	Cryptorchid (*n* = 33)	*p*-Value*
Maternal age (years)	29.8 (23.0 to 42.6)	29.8 (25.8 to 39.2)	0.979	27.7 (19.9 to 38.5)	29.7 (21.5 to 39.9)	0.306
BMI (kg/m^2^)^a^	22.9 (19.1 to 31.6)	23.0 (17.9 to 30.5)	0.997	22.8 (18.4 to 35.6)	22.0 (18.2 to 27.9)	0.106
Diabetes (yes/no)	1/35	1/28	0.877	0/32	4/29	0.044
Smoking (yes/no)	9/27	7/22	0.937	4/28	6/27	0.529
Parity (no.)						
1	28	20	0.451	19	17	0.405
2	4	5		9	9	
≥ 3	4	4		4	7	
Gestational age (days)	284 (253 to 297)	278 (219 to 296)	0.039	280 (160 to 296)	281 (249 to 296)	0.768
WGA (%)	1.89 (−23.5 to 35.6)	0.62 (−29.7 to 30.5)	0.687	−1.59 (−21.9 to 20.0)	1.17 (−30.8 to 27.3)	0.451
Birth weight (kg)	3.73 (2.65 to 5.15)	3.52 (1.31 to 4.85)	0.139	3.46 (2.86 to 4.46)	3.83 (2.51 to 4.66)	0.397
Birth length (cm)	53 (48 to 57)	52 (35 to 59)	0.264	51 (47 to 55)	52 (46 to 55)	0.826
Cryptorchidism at 3 months (no.)	—	4	—	—	25	—
Prematurity (no.)^b^	1	4	—	2	1	—
SGA (no.)	1	2	—	0	1	—
Infant blood samples (no.)	21	24		25	26	

WGA, weight for gestational age.

a Maternal prepregnancy BMI.

b < 259 days of gestation.

* Difference between cryptorchid boys and controls.

**Table 2:** Population characteristics [median (2.5th–97.5th percentiles)] for placentas.

	Denmark			Finland		
Characteristic	Control (*n* = 129)	Cryptorchid (*n* = 39)	*p*-Value*	Control (*n* = 56)	Cryptorchid (*n* = 56)	*p*-Value*
Maternal age (years)	31.0 (22.7 to 38.5)	29.5 (25.7 to 45.7)	0.723	28.1 (20.6 to 38.1)	29.1 (20.1 to 42.2)	0.349
BMI (kg/m^2^)^a^	22.1 (18.2 to 35.5)	21.5 (17.8 to 36.1)	0.199	22.3 (17.8 to 31.7)	23.1 (17.8 to 37.5)	0.062
Diabetes (yes/no)	2/127	0/39	0.435	0/55	10/46	0.001
Smoking (yes/no)	32/91	11/28	0.788	9/47	4/48	0.793
Parity (no.)						
1	81	26	0.628	31	31	0.997
2	36	10		19	19	
≥ 3	12	3		6	6	
Gestational age (days)	283 (254 to 298)	276 (195 to 294)	0.001	280 (255 to 293)	280 (258 to 303)	0.940
WGA (%)	0.29 (−23.3 to 34.9)	−0.83 (−39.5 to 44.4)	0.921	−0.32 (−21.7 to 25.7)	−1.12 (−27.9 to 25.0)	0.895
Birth weight (kg)	3.63 (2.49 to 5.06)	3.45 (0.75 to 4.75)	0.038	3.54 (2.85 to 4.61)	3.63 (2.55 to 4.58)	0.807
Birth Length (cm)	53 (47 to 57)	52 (32 to 60)	0.230	51 (47 to 55)	51 (48 to 55)	0.955
Cryptorchidism at 3 months (no.)	—	8	—	—	33	—
Prematurity (no.)^b^	6	6	—	3	1	—
SGA (no.)	5	2	—	0	3	—
Infant blood samples (no.)	88	25		45	35	

WGA, weight for gestational age.

a Maternal prepregnancy BMI.

b < 259 days of gestation.

* Difference between cryptorchid boys and controls.

**Table 3:** Seven PBDEs, detectable in all breast milk samples from Danish and Finnish boys with and without cryptorchidism [median (2.5th–97.5th percentiles)].

	Denmark			Finland			Both countries		
PBDE congener (ng/g fat)	Control (*n* = 36)	Cryptorchid (*n* = 29)	*p*-Value*	Control (*n* = 32)	Cryptorchid (*n* = 33)	*p*-Value*	Control (*n* = 68)	Cryptorchid (*n* = 62)	*p*-Value*
Sum of 7 congeners	3.21 (1.09–9.07)	4.12 (1.34–18.78)	0.017	3.08 (1.04–29.17)	4.27 (1.43–56.30)	0.111	3.16 (1.08–21.47)	4.16 (1.39–51.62)	0.007
47	1.05 (0.45–3.63)	1.53 (0.34–11.7)	0.018	1.24 (0.4–15.20)	1.82 (0.65–38.90)	0.062	1.12 (0.42–12.87)	1.56 (0.45–33.13)	0.003
153	1.0 (0.31–3.35)	1.18 (0.64–3.14)	0.226	0.67 (0.22–2.97)	0.68 (0.27–3.63)	0.394	0.81 (0.28–3.08)	0.94 (0.33–3.35)	0.156
99	0.44 (0.07–1.58)	0.64 (0.07–2.31)	0.132	0.39 (0.13–5.94)	0.48 (0.09–13.10)	0.366	0.42 (0.10–3.19)	0.53 (0.09–10.48)	0.091
100	0.26 (0.10–0.81)	0.37 (0.10–1.98)	0.019	0.30 (0.12–1.42)	0.37 (0.11–5.18)	0.128	0.27 (0.10–1.37)	0.37 (0.11–4.65)	0.008
28	0.10 (0.03–0.29)	0.13 (0.04–0.74)	0.030	0.12 (0.03–2.44)	0.17 (0.05–0.68)	0.099	0.10 (0.03–2.19)	0.15 (0.05–0.71)	0.005
66	0.04 (0.01–0.25)	0.08 (0.01–0.26)	0.0001	0.03 (0.01–1.38)	0.032 (0.01–0.19)	0.451	0.03 (0.01–0.57)	0.05 (0.01–0.25)	0.002
154	0.04 (0.01–0.13)	0.09^a^ (0.00–0.18)	0.0001	0.04 (0.02–0.18)	0.04 (0.02–0.54)	0.536	0.04 (0.01–0.17)	0.05 (0.01–0.50)	0.001
Sum of all 14 (ng/L)	83.4 (29.2–396.9)	119.3 (34.6–757)	0.009	163.2 (17.7–1308.5)	119.2 (64.3–2657.4)	0.581	104.2 (26.1–1188.4)	119.3 (36.4–1785.1)	0.046

a One unmeasurable sample.

* Adjusted for maternal age, BMI, parity, and date of childbirth, for the combined data also for country of origin.

**Table 4:** Seven less-prevalent PBDEs in breast milk samples from Danish and Finnish boys with and without cryptorchidism.

	Denmark				Finland			
PBDE congener (ng/g fat)	Control (*n* = 36)	%	Cryptorchid (*n* = 29)	%	Control (*n* = 32)	%	Cryptorchid (*n* = 33)	%
Sum of 14 congeners	3.27 (1.11–9.12)	—	4.27 (1.34–19.10)	—	3.11 (1.04–29.5)	—	4.27 (1.43–56.44)	—
183	0.05 (0.0–0.58)	78	0.05 (0.0–0.23)	62	0.0 (0.0–0.17)	44	0.0 (0.0–0.49)	27
85	0.005 (0.0–0.05)	67	0.07 (0.0–0.22)	97	0.0 (0.0–0.12)	6	0.0 (0.0–0.08)	36
75	0.001 (0.0–0.01)	61	0.0 (0.0–0.01)	28	0.0 (0.0–0.06)	34	0.0 (0.0–0.03)	36
77	0.001 (0.0–0.03)	53	0.01 (0.0–0.04)	65	0.0 (0.0–0.12)	19	0.0 (0.0–0.02)	15
119	0.0 (0.0–0.01)	25	0.0 (0.0–0.01)	48	0.0 (0.0–0.02)	12	0.0 (0.0–0.01)	15
138	0.0 (0.0–0.02)	28	0.0 (0.0–0.03)	28	0.0 (0.0–0.01)	3	—	0

Concentrations are given as median (2.5th–97.5th percentiles). Percentages are of detectable samples. BDE-71 was not detected in any sample.

**Table 5:** PBDEs in placentas from Danish and Finnish boys with and without cryptorchidism [median (2.5th–97.5th percentiles)].

	Denmark				Finland			
PBDE congener (ng/g fat)	Control (*n* = 129)	%	Cryptorchid (*n* = 39)	%	Control (*n* = 56)	%	Cryptorchid (*n* = 56)	%
47	0.39 (0.19–1.64)	100	0.40 (0.20–2.17)	100	0.60 (0.12–6.19)	100	0.52 (0.12–4.07)	100
153	0.44 (0.20–1.21)	100	0.41 (0.21–0.98)	100	0.20 (0.08–0.92)	100	0.20 (0.10–0.53)	100
99	0.23 (0.10–0.99)	100	0.18 (0.13–0.68)	100	0.19 (0.04–1.57)	100	0.14 (0.04–1.20)	100
100	0.11 (0.05–0.40)	100	0.10 (0.06–0.56)	100	0.11 (0.03–0.79)	100	0.10 (0.04–0.68)	100
28	0.03 (0.01–0.06)	100	0.03 (0.01–0.40)	100	0.04 (0.01–0.52)	100	0.04 (0.01–0.12)	100
Sum of 5 congeners	1.28 (0.59–3.26)	100	1.13 (0.79–4.45)	100	1.16 (0.35–9.65)	100	1.05 (0.35–6.51)	100
66	0.0 (0.0–0.03)	29	0.0 (0.0–0.04)	38	0.01 (0.0–0.20)	64	0.01 (0.0–0.04)	62
154	0.0 (0.0–0.03)	39	0.0 (0.0–0.02)	5	0.01 (0.0–0.10)	79	0.0 (0.0–0.09)	34
183	0.0 (0.0–0.14)	26	0.0 (0.0–0.09)	10	—	0	0.0 (0.0–0.07)	5
85	0.0 (0.0–0.01)	2	—	0	0.0 (0.0–0.08)	25	0.0 (0.0–0.04)	34
75	(0.003)	1	—	0	—	0	—	0
77	—	0	—	0	0.0 (0.0–0.02)	4	—	0
119	0.0 (0.0–0.002)	3	—	0	—	0	(0.01)	2
138	—	0	—	0	0.0 (0.0–0.02)	2	—	0
Sum of 14 congeners	1.31 (0.61–3.31)		1.13 (0.79–4.48)		1.18 (0.35–9.89)		1.06 (0.36–6.67)	

BDEs 153, 99, 100, and 28 were detected in all samples. BDE-71 was not detected in any sample. Percentages are of detectable samples.

**Table 6:** Correlations between PBDE measurements in paired placenta and breast milk samples (ng/g fat) of 43 Danish and 43 Finnish boys [median (95% CI).

PBDE congener	Median placenta	Median milk	*r*	*p*-Value
28	0.03 (0.02–0.39)	0.12 (0.03–1.85)	0.73	0.0001
47	0.42 (0.19–2.84)	1.27 (0.45–14.7)	0.64	0.0001
66	0.0 (0.0–0.06)	0.04 (0.01–0.27)	0.31	0.004
71	0.0 (0.0–0.0)	0.0 (0.0–0.0)	—	—
75	0.0 (0.0–0.0)	0.0 (0.0–0.02)	0.21	0.057
77	0.0 (0.0–0.004)	0.0 (0.0–0.03)	0.30	0.006
85	0.0 (0.0–0.06)	0.0 (0.0–0.12)	−0.02	0.823
99	0.19 (0.06–1.30)	0.42 (0.09–5.27)	0.55	0.0001
100	0.11 (0.05–0.43)	0.29 (0.10–1.41)	0.74	0.0001
119	0.0 (0.0–0.0)	0.0 (0.0–0.01)	—	—
138	0.0 (0.0–0.0)	0.0 (0.0–0.02)	−0.05	0.664
153	0.32 (0.11–0.93)	0.85 (0.27–3.11)	0.81	0.0001
154	0.0 (0.0–0.09)	0.04 (0.01–0.18)	0.26	0.015
183	0.0 (0.0–0.18)	0.02 (0.0–0.38)	0.26	0.015
Sum	1.19 (0.59–6.11)	3.23 (1.16–27.6)	0.66	0.0001

Due to nondetectable levels, correlations could not be computed for BDE-71 and BDE-119.

## References

[b1-ehp0115-001519] Agency for Toxic Substances and Disease Registry (2004). Toxicological Profile for Polybrominated Biphenyls and Polybrominated Diphenyl Ethers (PBBs and PBDEs). www.atsdr.cdc.gov/toxprofiles/tp68.html.

[b2-ehp0115-001519] Alaee M, Arias P, Sjödin A, Bergman A (2006). An overview of commercially used brominated flame-retardants, their application, their use in different countries/regions and possible modes of release. Environ Int.

[b3-ehp0115-001519] Andersson A-M, Toppari J, Haavisto A-M, Petersen JH, Simell T, Simell O (1998). Longitudinal reproductive hormone profiles in infants: peak of inhibin B levels in infant boys exceeds levels in adult men. J Clin Endocrinol Metab.

[b4-ehp0115-001519] [Anonymous] (1986). Cryptorchidism: an apparent substantial increase since 1960. John Radcliffe Hospital Cryptorchidism Study Group. BMJ.

[b5-ehp0115-001519] [Anonymous] (2005). Polybrominated diphenyl ether levels among United States residents: daily intake and risk of harm to the developing brain and reproductive organs. Integr Environ Assess Manag.

[b6-ehp0115-001519] Becher G, Nicolaysen T, Thomsen C (2001). Interlaboratory Comparison on Dioxins in Food 2001. Final Report 4.

[b7-ehp0115-001519] Betts K (2002). Rapidly rising PBDE levels in North America. Environ Sci Technol.

[b8-ehp0115-001519] Bi X, Qy W, Sheng G, Zhang W, Mau B, Chen D (2006). Polybrominated diphenyl ethers in South China, maternal and fetal blood and breast milk. Environ Pollut.

[b9-ehp0115-001519] Boisen KA, Kaleva M, Main KM, Virtanen HE, Haavisto A-M, Schmidt IM (2004). Difference in prevalence of congenital cryptorchidism in infants between two Nordic countries. Lancet.

[b10-ehp0115-001519] Ceccatelli R, Faass O, Schlumpf M, Lichtensteiger W (2006). Gene expression and estrogen sensitivity in rat uterus after developmental exposure to the polybrominated diphenyl ether PBDE-99 and PCB. Toxicology.

[b11-ehp0115-001519] Chao HR, Wang SL, Lee WJ, Wang YF, Papke O (2007). Levels of polybrominated diphenyl ethers (PBDEs) in breast milk from central Taiwan and their relation to infant birth outcome and maternal menstruation effects. Environ Int.

[b12-ehp0115-001519] Damgaard IN, Jensen TK, Petersen JH, Skakkebæk NE, Toppari T, Main KM (2007). Cryptorchidism and maternal alcohol consumption during pregnancy. Environ Health Perspect.

[b13-ehp0115-001519] Damgaard IN, Skakkebaek NE, Toppari J, Virtanen HE, Shen H, Schramm KW (2006). Persistent pesticides in human breast milk and cryptorchidism. Environ Health Perspect.

[b14-ehp0115-001519] Darnerud PO, Eriksen GS, Johannesson T, Larsen PB, Viluksela M (2001). Polybrominated diphenyl ethers: occurrence, dietary exposure and toxicology. Environ Health Perspect.

[b15-ehp0115-001519] Darnerud PO, Risberg S (2006). Tissue localization of tetra- and pentabromodiphenyl ether congeners (BDE-47, 85 and -99) in perinatal and adult C57BL mice. Chemosphere.

[b16-ehp0115-001519] Eslami B, Koizumi A, Ohta S, Inoue K, Aozasa O, Harad K (2006). Large-scale evaluation of the current level of polybrominated diphenyl ethers (PBDEs) in breast milk from 13 regions of Japan. Chemosphere.

[b17-ehp0115-001519] Hardell L, van Bavel B, Lindstrom G, Eriksson M, Carlberg M (2006). *In utero* exposure to persistent organic pollutants in relation to testicular cancer risk. Int J Androl.

[b18-ehp0115-001519] Inoue K, Harada K, Takenaka K, Uehara S, Kono M, Shimizu T (2006). Levels and concentration of polychlorinated biphenyls and polybrominated diphenyl ethers in serum and breast milk in Japanese mothers. Environ Health Perspect.

[b19-ehp0115-001519] Jaraczewska K, Lulek J, Cobaci A, Voorspoels S, Kaluba-Skotarczak A, Drews K (2006). Distribution of polychlorinated biphenyls, organochlorine pesticides and polybrominated diphenyl ethers in human umbilical cord serum, maternal serum and milk from Wielkopolska region, Poland. Sci Total Environ.

[b20-ehp0115-001519] Jensen AA, Slorach SA (1991). Chemical Contaminants in Human Milk.

[b21-ehp0115-001519] Kalantzi OL, Martin FL, Thomas GO, Alcock RE, Tang HR, Drury SC (2004). Different levels of polybrominated diphenyl ethers (PBDEs) and chlorinated compounds in breast milk from two U.K. regions. Environ Health Perspect.

[b22-ehp0115-001519] Kiviranta H, Ovaskainen ML, Vartiainen T (2004). Market basket study on dietary intake of OCDD/Fs, PCBs and PBDEs in Finland. Environ Int.

[b23-ehp0115-001519] Koppe JG, Bartonova A, Bolte G, Bistrup ML, Busby C, Butter M (2006). Exposure to multiple environmental agents and their effect. Acta Paediatr (Suppl).

[b24-ehp0115-001519] Kuriyama SN, Talsness CE, Grote K, Chahoud I (2005). Developmental exposure to low dose PBDE-99: effects on male fertility and neurobehaviour in rat offspring. Environ Health Perspect.

[b25-ehp0115-001519] Law RJ, Allchin CR, de Boer J, Covaci A, Herzke D, Lepom P (2006). Levels and trends of brominated flame-retardants in the European environment. Chemosphere.

[b26-ehp0115-001519] Legler J, Brouwer A (2003). Are brominated flame-retardants endocrine disrupters?. Environ Int.

[b27-ehp0115-001519] Lilienthal H, Hack A, Roth-Härer A, Wichert Grande S, Talsness CE (2006). Effects of developmental exposure to 2,2′4,4′,5-pentabromodiphenyl ether (PBDE-99) on sex steroids, sexual development, and sexually dimorphic behavior in rats. Environ Health Perspect.

[b28-ehp0115-001519] Lind Y, Darnerud PO, Atuma S, Aune M, Becker W, Bjerselius R (2003). Polybrominated diphenyl esters in breast milk from Uppsala County, Sweden. Environ Res.

[b29-ehp0115-001519] Main KM, Mortensen GK, Kaleva M, Boisen K, Damgaard I, Chellakooty M (2006a). Human breast milk contamination with phthalates and alterations of endogenous reproductive hormones in 3-month-old infants. Environ Health Perspect.

[b30-ehp0115-001519] Main KM, Schmith IM, Skakkebæk NE (2000). A possible role for reproductive hormones in newborn boys: progressive hypogonadism without the postnatal testosterone peak. J Clin Endocrinol Metab.

[b31-ehp0115-001519] Main KM, Toppari J, Suomi AM, Kaleva M, Chellakooty M, Schmidt IM (2006b). Larger testes and higher inhibin B levels in Finnish than in Danish newborn boys. J Clin Endocrinol Metab.

[b32-ehp0115-001519] Marsál K, Persson PH, Larsen T, Lilja H, Selbing A, Sultan B (1996). Intrauterine growth curves based on ultrasonically estimated fetal weights. Acta Paediatr.

[b33-ehp0115-001519] Mazdai A, Dodder NG, Anernathy MP, Hites RA, Bigsby RM (2003). Polybrominated diphenyl ethers in maternal and fetal blood samples. Environ Health Perspect.

[b34-ehp0115-001519] McDonald TA (2002). A perspective on the potential health risks of PBDEs. Chemosphere.

[b35-ehp0115-001519] Meerts IA, Letcher RJ, Hoving S, Marsh G, Bergman A, Lemmen JG (2001). *In vitro* estrogenicity of polybrominated diphenyl ethers, hydroxylated PBDEs, and polybrominated bisphenol A compounds. Environ Health Perspect.

[b36-ehp0115-001519] Meironyte D, Noren K, Bergman A (1999). Analysis of polybrominated diphenyl ethers in Swedish human milk. A time-related trend study, 1972–1997. J Toxicol Environ Health A.

[b37-ehp0115-001519] Ortiz-Olaya N, Flores ME, De Santiago S (1996). Significance of lipid consumption during lactation. Rev Invest Clin.

[b38-ehp0115-001519] Pihkala J, Hakala T, Voutilainen P, Raivio K (1989). Characteristic of recent fetal growth curves in Finland. Duodecim.

[b39-ehp0115-001519] Sharpe RM (2006). Pathways of endocrine disruption during male sexual differentiation and masculinization. Best Pract Res Clin Endocrinol Metab.

[b40-ehp0115-001519] Shen H, Main KM, Kaleva M, Virtanen H, Haavisto AM, Skakkebæk NE (2005). Prenatal organochlorine pesticides in placentas from Finland: exposure of male infants born during 1997–2001. Placenta.

[b41-ehp0115-001519] Shen H, Main KM, Virtanen HE, Damgaard IN, Haavisto AM, Kaleva M (2007). From mother to child: investigation of prenatal and postnatal exposure to persistent bioaccumulating toxicants using breast milk and placenta biomonitoring. Chemosphere.

[b42-ehp0115-001519] Shen H, Virtanen HE, Main KM, Kaleva M, Andersson AM, Skakkebæk NE (2006). Enantiomeric rations as an indicator of exposure processes for persistent pollutants in human placentas. Chemosphere.

[b43-ehp0115-001519] Sjödin A, Patterson DG, Bergman A (2003). A review on human exposure to brominated flame-retardants—particularly polybrominated diphenyl ethers. Environ Int.

[b44-ehp0115-001519] Skakkebæk NE, Rajpert-De Meyts E, Main KM (2001). Testicular dysgenesis syndrome: an increasingly common developmental disorder with environmental aspects. Hum Reprod.

[b45-ehp0115-001519] Småstuen Haug L, Becher G (2004). Interlaboratory Comparison on Dioxins in Food 2004. Fifth Round of An International Study.

[b46-ehp0115-001519] Småstuen Haug L, Becher G (2005). Interlaboratory Comparison on Dioxins in Food 2005. Sixth Round of An International Study.

[b47-ehp0115-001519] Staskal DF, Diliberto JJ, Birnbaum LS (2006). Disposition of BDE 47 in developing mice. Toxicol Sci.

[b48-ehp0115-001519] Stoker TE, Cooper RL, Lambright VS, Wilson F, Gray LE (2005). *In vivo* and *in vitro* anti-androgenic effects of DE-71, a commercial polybrominated diphenyl ether (PBDE) mixture. Toxicol Appl Pharmacol.

[b49-ehp0115-001519] Stoker TE, Laws SC, Crofton KM, Hedge JM, Ferrell JM, Cooper RL (2004). Assessment of DE-71, a commercial polybrominated diphenyl ether (PBDE) mixture in the EDSP male and female pubertal protocols. Toxicol Sci.

[b50-ehp0115-001519] Strandman T, Koistinen J, Variainen T (2000). Levels of some polybrominated diphenyl ethers (PBDEs) in placenta and human milk. Organohalogen Compounds.

[b51-ehp0115-001519] Suomi A-M, Main KM, Kaleva M, Schmidt IM, Chellakooty M, Virtanen HE (2006). Hormonal changes in 3-month-old cryptorchid boys. J Clin Endocrinol Metab.

[b52-ehp0115-001519] Talsness CE, Shakibaei N, Kuriyama SN, Grande SW, Sterner-Kock A, Schnitker P (2005). Ultra structural changes observed in rat ovaries following *in utero* and lactational exposure to low doses of a polybrominated flame retardant. Toxicol Lett.

[b53-ehp0115-001519] Toppari J (2003). Physiology and disorders of testicular descent. Endocr Dev.

[b54-ehp0115-001519] Virtanen HE, Tapanainen AE, Kaleva MM, Suomi AM, Main KM, Skakkebæk NE (2006). Mild gestational diabetes as a risk factor for congenital cryptorchidism. J Clin Endocrinol Metab.

[b55-ehp0115-001519] Waliszewski SM, Aquirre AA, Infanzon RM, Silva CS, Siliceo J (2001). Organochlorine pesticide levels in maternal adipose tissue, maternal blood serum, umbilical blood serum, and milk from inhabitants of Veracruz, Mexico. Arch Environ Contam Toxicol.

[b56-ehp0115-001519] Watanabe I, Tatsukawa R (1987). Formation of brominated dibenzofurans from the photolysis of flame-retardant decabromodiphenyl ether in hexane solution by UV and sunlight. Bull Environ Contam Toxicol.

[b57-ehp0115-001519] World Medical Association (2000). Declaration of Helsinki: Ethical principles for medical research involving human subjects. Bull Med Eth.

